# Oxygen—A Critical, but Overlooked, Nutrient

**DOI:** 10.3389/fnut.2019.00010

**Published:** 2019-02-12

**Authors:** Paul Trayhurn

**Affiliations:** ^1^Clore Laboratory, University of Buckingham, Buckingham, United Kingdom; ^2^Obesity Biology Unit, University of Liverpool, Liverpool, United Kingdom

**Keywords:** adipocyte, adipose tissue, hypoxia, hypoxia-inducible factor-1 (HIF-1), mitochondria, oxygen deficiency, respiration

## Abstract

Gaseous oxygen is essential for all aerobic animals, without which mitochondrial respiration and oxidative phosphorylation cannot take place. It is not, however, regarded as a “nutrient” by nutritionists and does not feature as such within the discipline of nutritional science. This is primarily a consequence of the route by which O_2_ enters the body, which is via the nose and lungs in terrestrial animals as opposed to the mouth and gastrointestinal tract for what are customarily considered as nutrients. It is argued that the route of entry should not be the critical factor in defining whether a substance is, or is not, a nutrient. Indeed, O_2_ unambiguously meets the standard dictionary definitions of a nutrient, such as “*a substance that provides nourishment for the maintenance of life and for growth*” (Oxford English Dictionary). O_2_ is generally available in abundance, but deficiency occurs at high altitude and during deep sea dives, as well as in lung diseases. These impact on the provision at a whole-body level, but a low pO_2_ is characteristic of specific tissues includings the retina and brain, while deficiency, or overt hypoxia, is evident in certain conditions such as ischaemic disease and in tumours - and in white adipose tissue in obesity. Hypoxia results in a switch from oxidative metabolism to increased glucose utilisation through anaerobic glycolysis, and there are extensive changes in the expression of multiple genes in O_2_-deficient cells. These changes are driven by hypoxia-sensitive transcription factors, particularly hypoxia-inducible factor-1 (HIF-1). O_2_ deficiency at a whole-body level can be treated by therapy or supplementation, but O_2_ is also toxic through the generation of reactive oxygen species. It is concluded that O_2_ is a critical, but overlooked, nutrient which should be considered as part of the landscape of nutritional science.

## Introduction

There is a general understanding of what constitutes the field of nutritional science. At its core, the discipline encompasses the provision of macro- and micro-nutrients, how they are processed in order to meet the requirements of the individual (whether humans or other species) for maintenance—and at certain points in the life cycle, for growth and development. From a biomedical perspective, the focus is with the consequences of under– or over-provision with regard to health and the prevention of disease. Most nutritional scientists understand macronutrients to mean proteins, carbohydrates, and lipids, each of which include multiple molecular entities. Alcohol may also be classified as a macronutrient, and in some cultures makes a significant contribution to total energy intake.

There are, however, two major nutrients which are essentially, or entirely, ignored in nutrition, not least in textbooks of the subject, and water is the most obvious example. Water is, of course, contained within most foods and is released during digestion; it is also obtained as a by-product of multiple metabolic processes. There is nonetheless, a distinct requirement for additional water, particularly in hot environments and in situations such as during extreme exertion when the losses are high. Indeed, the provision of (clean) water for direct consumption is a critical determinant of the health and survival of man and other animals. Water is consumed as such both during and between meals, and enters the body in a manner similar to that of other nutrients.

The other forgotten, though quantitatively major nutrient, is diatomic oxygen. O_2_ has, however, rarely been considered as a nutrient in the customary sense of the term, certainly in the case of humans and other higher animals; this is despite earlier arguments that it should be so considered, though the proponents were equivocal (1). Indeed, its classification as a nutrient would be regarded as controversial by many nutritionists. In the present article, the proposition that O_2_ is unambiguously a nutrient (1, 2) and should be included within the landscape of nutritional science is developed.

## Definitions—What Is a Nutrient?

Whether O_2_, a gas, is a macro (in the sense of quantity) nutrient and part of the remit of nutrition depends, of course, on the meaning of the word “nutrient”. There is a commonplace sense of what constitutes a nutrient—as something that is eaten within food and which is necessary for bodily maintenance and good health. However, dictionaries have, as would be expected, clear definitions of the term. In the Oxford Dictionary of English, 2nd edition (2005)—the Oxford dictionaries being the “bible” of the English language—a nutrient is defined as “*a substance that provides nourishment for the maintenance of life and for growth*”. Its American equivalent, Webster's, is perhaps even more precise in its definition, a nutrient being “*any substance or matter that is needed for the life and growth of living things”*. Other dictionaries offer similar definitions (e.g., Collins English Dictionary: “*any substance that nourishes an animal”*), although the word “food” is sometimes included by those that are medical in focus.

It is clear from these definitions that O_2_, as well as water, is unambiguously a nutrient. In the case of water, its inclusion would not be considered controversial, given that it is consumed via the mouth and is absorbed from the gastrointestinal tract similar to those substances that are classically regarded as nutrients. The provision of O_2_ is, of course, fundamentally different in that it is obtained from the ambient air as a gas and delivered through the nose and lungs (or gills in the case of aquatic animals). It is this route of entry into the body which is the primary reason why O_2_ is not regarded as a nutrient in humans and other higher animals, and is absent from nutritional discourse. Indeed, reference to O_2_ in a nutritional context is invariably limited to metabolic rate and energy expenditure with respect to energy balance and RQ (respiratory quotient).

It is argued that the actual route of entry—gastrointestinal tract or lungs/gills—should not be considered a defining characteristic of a substance as a nutrient, but rather the functions and essentiality as delineated in the dictionary definitions above (Table 1). With considerable insight, the Polish alchemist Michael Sendivogius wrote as far back as 1604 that “Man was created of the Earth, and lives by virtue of the air; *for there is in the air a secret food of life*….” (my italics) (3). This was, of course, well-before the formal discovery of O_2_, though Sendivogius in effect hypothesised its existence. The discovery of O_2_ as such in the latter half of the 18th century is variously credited to the English chemist Joseph Priestley, the Swedish apothecary Carl Scheele, and the French chemist Antoine Lavoisier, each contributing in different respects.

**Table 1:** Characteristics of oxygen as compared to what are normally regarded as nutrients.

	**Oxygen**	**Classical nutrients**
Obtained from	The ambient air (surrounding water in fish)	Foods in the diet
Route of entry	Nose and lungs (gills in fish)	Mouth and gastrointestinal tract
Frequency of provision	Essentially constant	Generally periodic
Processing	No prior processing	Requires processing by digestion
Transportation	Transported directly by a specific transporter (*haemoglobin*)	In some cases transported by specific transporters
Storage	Storage is minimal–essentially only in muscle (*myoglobin*) in some species for local use	May be stored for future use (e.g., fatty acids as triacyglycerols in adipose tissue, and glucose as glycogen in liver)
Deficiency	Available in abundance–but deficiency at high altitudes, during deep diving, in specific ecological niches and in lung diseases. Major cellular and molecular adaptations to reduced levels	Well-recognised deficiency diseases for most nutrients
Toxicity	Some toxicity at normal levels	Excess can be toxic for specific nutrients (e.g., vitamin A, selenium)
RDA	Potentially-to maintain energy balance and to meet requirements for growth, pregnancy and lactation	Yes

The word oxygen is derived from the Greek for “acid-former” (*oxys*—sharp, sour and *genes*—producer), which referred to the mistaken view that it was necessary for the formation of acids. The name was first coined by Lavoisier (French, *oxygène*) who showed that combustion was the reaction of O_2_ with carbon and other substances.

## Oxygen and the Origins of Life

It is an axiom that life on Earth as we know it is dependent on the presence of an atmosphere containing O_2_. Gaseous oxygen has been detected elsewhere in the universe, its presence in the atmosphere of distant exoplanets providing a potential biomarker for life (4–6). When the Earth first formed 4.54 billion years ago through accretion from the solar nebula, the primordial atmosphere was devoid of O_2_—there was no free O_2_ (7, 8). The diatomic oxygen that was present was essentially locked up in rocks and in water, and some 4 billion years ago the atmosphere was thought to have contained O_2_ at just one part in a million (8, 9). The earliest single celled organisms existed under anoxic conditions and were superseded by cyanobacteria, which appeared up to 3 billion years or more ago—and, importantly, generated O_2_ through photosynthesis (9). Microorganisms, such as those harboured in deep sea hydrothermal vents, are able to survive, indeed flourish, under anoxic conditions through sulphur respiration—anaerobic respiration with sulphur (10, 11).

Approximately 2.45 billion years ago O_2_ began to appear in the atmosphere in significant quantities at what is termed the “Great Oxidation Event” (actually a protracted process) (9, 12, 13). O_2_ levels gradually increased, and probably then fell, until around 700 million years ago when a further sharp increase occurred (12). The level of O_2_ in the atmosphere is believed to have hovered at around the current 21% since ~600 million years ago (9). While O_2_ can be toxic in many respects, as discussed in a later section, its rise was critical to the development of multi-cellular organisms and the physiological complexity that this implies. The earliest known fossils of a eukaryote, from which multi-cellular organisms evolved, date from at least 2 billion years ago (14). In eukaryotes and early multi-cellular organisms requiring O_2_, uptake occurs by direct transfer across the cell membrane in essentially the same manner as other nutrients prior to the development of specialised digestive and respiratory organs.

The ready availability of O_2_ had a profound effect on the metabolic opportunities for an organism and the consequent systems that developed. The mitochondrion, through respiration and oxidative phosphorylation, is a potent example of a cellular organelle whose evolution resulted in major new metabolic processes. The most widely accepted view on the origin of mitochondria is the endosymbiotic hypothesis which proposes that mitochondria were originally prokaryotic cells (14, 15). These prokaryotes were able to undertake oxidative processes that early eukaryotic cells could not perform, and they subsequently became endosymbionts living within the eukaryote cell structure.

## Provision and Delivery of O_2_

Animals are constant metabolisers, whether they are poikilotherms or homeotherms, and this is so even in those species that undergo periods of hibernation, aestivation or torpor (albeit at a reduced rate of metabolism). However, most nutrients are obtained in higher animals on an intermittent basis, such species being periodic feeders—whether in the form of distinct meals or through frequent foraging. Foods, entering through the mouth, are generally complex structures and the nutrients that they contain are not immediately available. Instead, they require release through digestion and are subsequently absorbed from the gastrointestinal tract, a process that may involve specific transporters.

In simple organisms, O_2_ is obtained in a manner similar to that of other nutrients—by absorption across the cell membrane—while in complex organisms it is fundamentally different. The evolution of specialised organs has resulted in the development of a respiratory system for the delivery of O_2_, differentiating it sharply from the route by which all other nutrients are provided through the digestive system (Table 1). In marked contrast to nutrients obtained via the mouth and gastrointestinal tract, the requirement for O_2_ is virtually continuous in higher animals; as is well-recognised, the absence of O_2_ results in death within a very few minutes in humans and many other higher species. This reflects both the constant metabolic need for O_2_ together with the absence of any significant storage. There is some limited storage, however, in skeletal muscle for local use through binding to the iron-containing protein myoglobin, but this is primarily a feature of marine animals such as whales, which experience apnoea during diving and where the haem protein is present in relative abundance (16).

On entering the lungs, O_2_ passes into the alveoli which as highly vascularised sacs enable the rapid movement of the gas by simple diffusion, first across the alveolar epithelium and then the endothelial cells of the alveolar capillaries. Once in the circulation, O_2_ binds to haemoglobin in the erythrocytes and is immediately transported to tissues (17). Modifications to this route of entry occur through the presence of gills in aquatic species, while in lower animals simpler systems for obtaining O_2_ are evident.

The presence of haemoglobin as a specific carrier for O_2_ has some parallels with the transport and delivery of a number of other nutrients. There are selective carriers for the movement of nutrients from the lumina of the gastrointestinal tract—such as the Na+-dependent glucose transporter (SGLT1) to transfer glucose (18, 19), and several amino acid transporters to transfer various amino acids (19, 20). Once across the gastrointestinal wall, from mucosal to serosal side, nutrients move to their immediate sites of action or to storage organs for subsequent use. Storage occurs particularly in the liver and skeletal muscle for glucose as glycogen, and in white adipose tissue depots for the sequestration of fatty acids as triacylglycerols (19). In some cases, carrier proteins are involved in the transport of nutrients to their storage site, such as transferrin for the transport of iron to the bone marrow (21). Specific carriers, analogous to haemoglobin, also transport a number of nutrients to the tissues where they are required once released from storage, examples including retinol binding protein for retinol (21, 22) and plasma lipoproteins in the case of lipids (19, 23).

## Metabolic Function of O_2_

Despite O_2_ not being recognised as a nutrient in the context of nutritional science and in relation to whole-body physiology, it is frequently described as such in studies at a cellular level [e.g., (1, 24)]. The central role of O_2_ as a nutrient is in mitochondrial respiration, acting as an electron acceptor thereby enabling ATP to be formed through oxidative phosphorylation. This process is fundamental to aerobic organisms, with the oxidation of glucose and fatty acids requiring the continuous provision of O_2_. Several core metabolic pathways are central to mitochondrial oxidative phosphorylation—glycolysis, glycogenolysis, lipolysis, and the tricarboxylic acid (Krebs) cycle (19).

There are differences between cell types in the number of mitochondria that are present, as well as in the relative development of their cristae structure, according to the level of respiration and oxidative phosphorylation that is functionally required. White adipocytes, for example, have moderate numbers of mitochondria which contain limited cristae, with most of the volume of these cells being due to the lipid droplet (25, 26). Brown adipocytes, in marked contrast, contain large numbers of mitochondria with a highly developed and dense cristae structure, especially in rodents adapted to cold environments when maximum non-shivering thermogenesis is required (25, 26). In these circumstances, brown fat mitochondria utilise substantial amounts of O_2_ in order to sustain the oxidation of fatty acids and other substrates at high rates, with ATP synthesis being bypassed through a proton leakage pathway regulated by UCP1 (uncoupling protein-1) (27).

## Deficiency of Oxygen

The partial pressure of O_2_ is highest at sea level, but falls with altitude leading to a decrease in the amount available. Altitude is one of the several environmental situations that result in a reduction in the availability of O_2_. Animals, including humans, that habitually live at high elevations have evolved distinct physiological adaptations which allow them to adapt to the relatively hypoxic conditions. Another environmental circumstance in which O_2_ deprivation occurs, albeit on a short-term basis, is that experienced by aquatic mammals such as whales during deep sea dives.

Even at sea level, a marked periodic lack of O_2_ is also evident in certain terrestrial species according to their precise ecological niche. Naked mole-rats (*Heterocephalus glaber*) are a potent example, these animals experiencing near anoxic conditions during prolonged periods in their subterranean burrows (28). The ability of naked mole-rats to withstand sustained anoxia is suggested as being due to the utilisation of fructose as a fuel for glycolysis, through high levels of the GLUT5 fructose transporter and of ketohexokinase, enabling the key glycolytic regulatory enzyme phosphofructokinase to be bypassed (28). Some ectothermic vertebrates, such as the American freshwater turtle and crucian carps, exhibit extreme capacities to withstand a low O_2_ tension, being able to survive for months under what is effectively complete anoxia (29).

These examples of O_2_ deprivation relate to specific environmental and ecological conditions to which particular species are exposed and effect provision of the macronutrient in whole-animal terms. A fall in the availability of O_2_ to the body as a whole can also occur in certain disease states, primarily disorders of the lungs (30). There are several major human lung diseases, some of which are often given the collective name of chronic obstructive pulmonary disease, that lead to impairments in O_2_ delivery; these include emphysema, pulmonary fibrosis and cystic fibrosis (31). Such conditions result in a chronic insufficiency of O_2_, but there are also situations in which there is an acute, periodic lack as in obstructive sleep apnoea (32).

The O_2_ tension of inspired air at sea level is 160 mmHg (21.3 kPa) while in alveolar blood it is around 104 mmHg (13.9 kPa), the level at which it leaves the lungs (7). The general level of tissue oxygenation is rather lower at some 40–50 mmHg (5.33–6.67 kPa); thus, even the “normal” tissue pO_2_ is in effect hypoxic relative to alveolar blood and ambient air at sea level (7, 33). However, a substantially lower O_2_ tension is evident in several tissues, including the spleen, thymus, retina and regions of the brain (which is a substantial consumer of O_2_) with a pO_2_ of 16 (2.13 kPa), 10 (1.33 kPa), 2–25 (0.27–3.33 kPa) and 0.4–8 mmHg (0.05–1.07 kPa), respectively (see Table 2) (34–36). Tumours are also markedly hypoxic, with pO_2_ values ranging from 1 to 10 mmHg (0.13–1.13 kPa) - and the centre of solid tumours may be essentially anoxic (7, 33, 34). These examples of low pO_2_ in certain tissues generally reflect limited vascularisation, as in the retina and in tumours, and the consequent distance that O_2_ has to travel to the cells where it is required. The diffusion distance of O_2_ is generally considered to be of the order of 100–200 μm – the size of large white adipocytes (38) - but there are reports of a pO_2_ close to zero at only 100 μm from the capillaries (7, 39).

**Table 2:** Oxygen level (pO_2_) in ambient air and in selected tissues.

**Tissue**	**pO_**2**_ in mmHg (References)**
Ambient (inspired) air at sea level	160 (7)
Alveolar blood	104 (7)
General tissue oxygenation	40–50 (7, 33)
Thymus	10 (34)
Spleen	16 (34)
Retina	2–25 (35)
Brain	0.4–8 (36)
Tumours	1–10 (7)
White adipose tissue-lean mice	47.9 (37)
White adipose tissue-obese mice	15.2 (37)

There are other pathological conditions, in addition to tumours, in which overt tissue hypoxia is evident. These include psoriasis and ischaemic disorders, and at the site of wounds during healing (7, 40, 41). A further example is with white adipose tissue depots in obesity, at least in rodents (41). The pO_2_ of white fat in lean mice is around 45–50 mmHg (6.00–6.67 kPa), similar to the general level of tissue oxygenation, but in obese mice it is between 2- and 3.5-fold lower at ~15 mmHg (2.00 kPa), as assessed with needle-type fibre optic O_2_ sensors (37, 42–44). This reduction is evident in genetically obese (*ob/ob*) mice and in mice made obese through the provision of a high fat diet (37, 42, 43, 45). The functional implications of this marked reduction in pO_2_ in white fat depots in obesity is considered in a later section.

## Cellular and Molecular Adaptations to Oxygen Deficiency

Cells have highly developed systems for detecting O_2_ levels and for adapting to deficiencies. O_2_ is sensed at the cell membrane by specific ion channels, particularly K+ channels (46–48). Within the cell, there are a series of transcription factors which are responsive to O_2_ and which drive the molecular adaptations to hypoxia. These include NFκB and CREB (cAMP response element-binding protein) (49), but the central transcription factors sensitive to hypoxia are the HIFs (hypoxia-inducible factors). There are three HIFs, of which HIF-1 is considered the most significant—and certainly the most widely studied (50–52).

HIF-1 is a heterodimer, the subunits being HIF-1α and HIF-1β. HIF-1β is constitutively expressed, and although HIF-1α is constantly synthesised it is rapidly degraded under normal cellular levels of O_2_ (7, 52–54). The mechanism by which HIF-1α is degraded has been extensively studied; in essence, degradation is through the 26S proteosomal system and involves the hydroxylation of HIF-1α. The HIF-1α subunit is stabilised when the O_2_ level is low, enabling the functional dimeric HIF-1 transcription factor to be produced.

The other HIFs - HIF-2 and HIF-3 - are formed by the dimerisation of HIF-1β with HIF-2α and HIF-3α, respectively (52, 55). These HIFs appear to be more limited in their action than HIF-1, particularly HIF-3, and are more tissue specific. HIF-1 regulates the transcription of in excess of 100 genes, these encoding proteins involved in several distinct cellular and metabolic systems (24, 40, 53). Importantly, enzymes and other proteins associated with glucose utilisation and glycolysis are regulated by HIF-1. Amongst the genes related to glycolysis that are up-regulated in response to hypoxia, though not necessarily by a HIF-1 dependent mechanism, are *GPI* (glucose-6-phosphate isomerase), *HK2* (hexokinase 2) and *PFKP* (phosphofructokinse platelet). There is also up-regulation by hypoxia of the expression of the gene encoding GLUT1, the facilitative glucose transporter responsible for basal glucose uptake, and this is widely used as a molecular marker of the cellular response to low pO_2_(40, 41).

Increases in the expression of genes associated with glucose uptake and utilisation reflect the augmentation of anaerobic glycolysis that occurs under conditions of low pO_2_. One of the consequences of greater glycolysis in hypoxia is a rise in the production of lactate, associated with increased expression of lactate transporters, MCT1 in particular in the case of adipocytes for example (56). There are also parallel changes in the expression of genes encoding mitochondrial enzymes and other proteins involved in respiration and oxidative phosphorylation, consequent to the reduction of these processes (57, 58). Again using white adipocytes as an example, the expression of genes such as *ATP5D* (ATP synthase, H+ transporting, mitochondrial F1 complex, delta subunit) and *COX4I1* (cytochrome *c* oxidase subunit 4, isoform 1) are down-regulated in hypoxia (41, 58).

## Oxygen Deficiency: The Example of White Adipose Tissue

In addition to changes in glucose utilisation, oxidative phosphorylation, and lipid oxidation, exposure to a low pO_2_ leads to alterations in the expression of multiple genes involved in several other pathways in white adipocytes, including cell death and cell-to-cell signalling and interaction (58, 59); indeed, the expression of ~1,300 genes is altered in adipocytes by hypoxia (58). These reflect general responses to hypoxic conditions, many of which are near universal, especially those linked to anaerobic glycolysis and oxidative phosphorylation. However, some of cellular and molecular adaptations to a low pO_2_ are specific to individual tissues, reflecting their particular physiological function.

**Figure 1:**
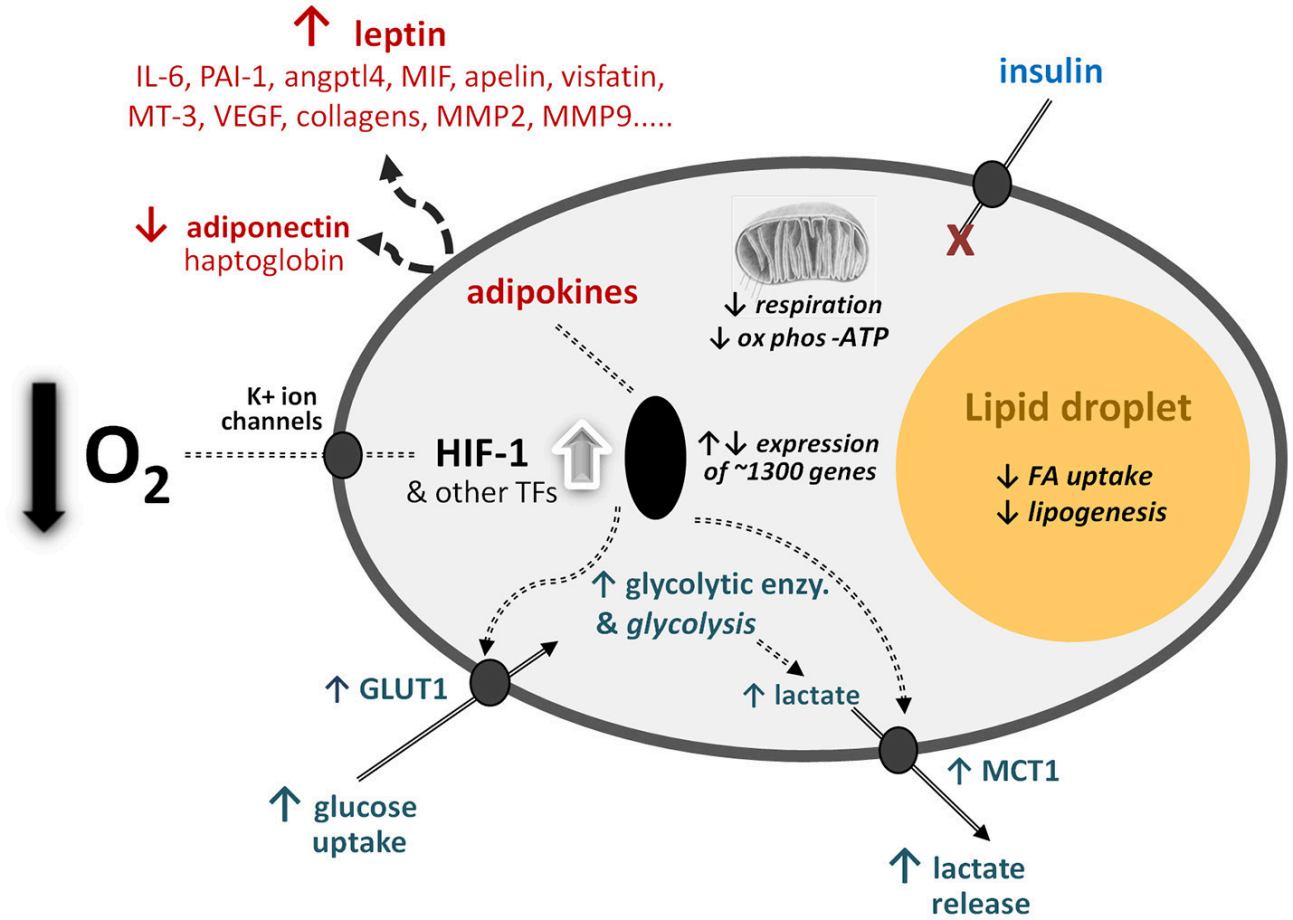
Schematic representation of some of the central cellular responses to hypoxia (oxygen deficiency) in white adipocytes. The figure illustrates adaptations that are universal to all cell types, particularly the increase in glucose utilisation through anaerobic glycolysis and the reduction in respiration and oxidative phosphorylation (ox phos). Adaptations that are specific to adipocytes are also shown, primarily those relating to lipid utilisation and the production of adipokines as key secretory proteins of these cell types; in some of the examples, such as MT-3 (metallothionein-3), only major changes at the gene expression level have been formally documented. angptl4, angiopoietin-like protein-4; enzy, enzyme; FA, fatty acid; GLUT1, facilitative glucose transporter 1; HIF-1, hypoxia-inducible factor-1; MCT1, monocarboxylate transporter-1; MIF, macrophage migration inhibitory factor; MMPs, matrix metalloproteinases; PAI-1, plasminogen activator inhibitor-1; TF, transcription factors (additional to HIF-1); VEGF, vascular endothelial growth factor. Adapted from (1).

A clear example comes from white adipose tissue (Figure 1), a tissue that has been a continuing focus in nutritional science. This was originally in relation to the storage of triacylglycerols as fuel, but subsequently as a consequence of the surge in the incidence of obesity. More recently, white adipose tissue has been recognised as a major endocrine and signalling organ, being implicated in a range of physiological functions—from the regulation of appetite and blood pressure to insulin sensitivity and the inflammatory response (41, 60). Much of this recent regulatory perspective on white fat centres on the multiplicity of protein factors—the adipokines—that are released by white adipocytes, and which number several hundreds (61). This has followed from the discovery of the hormone leptin, adipocytes being the major site of production of this pleiotropic endocrine factor (62).

Expression of the leptin (*LEP*) gene and secretion of the mature protein hormone are markedly stimulated by hypoxia (63), and indeed a low O_2_ tension induces preadipocytes to produce the hormone (64)—which they do not when incubated under so-called “normoxic” conditions (21% O_2_). Thus, exposure to O_2_ deficiency leads to a substantial increase in the production of a key adipocyte hormone. Another major adipocyte hormone, adiponectin, responds to hypoxia in the opposite direction, there being a reduction in the expression of the *ADIPOQ* gene and in the secretion of the encoded protein (63). This particular change would be expected to lead to a fall in insulin sensitivity and increased inflammation, given the insulin sensitising and anti-inflammatory actions of adiponectin (65–68).

Inflammation is one of the characteristics of adipose tissue as it enlarges in obesity, with recruitment of M1 macrophages and increased expression and release of a number of cytokines and chemokines, as well as of other inflammation-related factors (60, 69–71). Exposure of white adipocytes to hypoxic conditions, at least in cell culture, leads for example to increases in the expression of the *IL6, VEGF, PAI1, MIF, MMP2*, and *MMP9* genes, in addition to the *LEP* gene, as part of the inflammatory response (45, 63, 72). Inflammation in adipose tissue, driven by hypoxia (41, 51), is considered to underpin the development of the major obesity-associated diseases, particularly insulin resistance, type 2 diabetes and the metabolic syndrome (73, 74).

Most *in vitro* and cell culture studies on adipocytes, and indeed of other types of cell, use a protocol in which the response to 1 or 2% O_2_ is compared to that of a reference level of 21% O_2_. As noted previously, this represents an extreme with 21% O_2_ being in effect hyperoxia while 1% O_2_ is at the bottom end of normal tissue oxygenation. Despite being an extreme, it can be justified by enabling “proof of principle” to be explored, even though it may exaggerate the scale of the response to O_2_ deprivation. Studies in which human adipocytes are incubated with graded levels of O_2_ between 21 and 1% indicate that indeed the response is exaggerated (75). They also demonstrate that there is not a critical threshold at which adaptive changes to low pO_2_ occur, but rather that there is a graded “dose response” (75). This is true for a range of processes, including glucose uptake and lactate release, and to the expression and secretion of leptin, VEGF and other adipokines (75). Thus, adipocytes titre small changes in O_2_ tension (provision) across a range of metabolic systems and processes, and this is also likely to occur in other cell types.

## Oxygen Therapy and Toxicity

When there is a deficiency of O_2_, amelioration is possible through increasing its provision by O_2_ therapy, or supplementation. O_2_ therapy has its origins in the Pneumatic Institution founded in Bristol (UK) in 1799 by Thomas Beddoes, the aim of which was to explore the potential efficacy of the gas for the treatment of disease (76). Treatment now may be acute, as in medical emergencies such as for resuscitation, trauma and anaphylaxis, or chronic as in lung diseases, including chronic obstructive pulmonary disease and emphysema (30). Supplemental O_2_ is also utilised by mountaineers at high altitudes and in aircraft in the event of a fall in cabin pressure. These are, of course, relatively extreme conditions, and this is also the case with hyperbaric O_2_ therapy to treat decompression sickness in deep-sea divers.

There is a Janus-faced dimension to O_2_ in that in addition to being essential, it is toxic under certain conditions. For example, giving O_2_ to newborn premature babies can lead to blindness (77, 78), and O_2_ toxicity is a well-recognised risk of deep diving. At a molecular level, O_2_ is toxic through the formation of reactive oxygen species (ROS), these including peroxides, singlet oxygen, hydroxy radical and superoxide (79, 80). Although ROS are produced during normal metabolic functions involving O_2_, and have important signalling roles, their accumulation leads to cell damage and oxidative stress (79). Antioxidant defence mechanisms detoxify ROS, and these encompass enzymatic systems, in particular the superoxide dismutases, catalase and glutathione peroxidases(79, 80).

## Recommended ‘Dietary’ Allowance

The concept of the recommended dietary allowance (RDA) is based on the need to set standards for the intake of nutrients, and this has been a continuing concern for national agencies and for Governments. The terminology associated with the general concept has, however, become somewhat complex with different countries adopting differing terms—“dietary reference value” in the UK, “population reference intake” elsewhere in the European Union, and “dietary reference intake” in the USA and Canada, with many other countries still using RDA (81). Whichever term is employed, standards are available for almost all nutrients, including water.

The concept of a RDA (or equivalent) would appear problematic for O_2_ – not least because of the word “diet” in the term—and it is appropriate to ask whether in this case it is relevant. Under most circumstances O_2_ is freely available and essentially unlimited (with the exceptions described above), and is obtained without financial cost. Intake is tightly controlled by the respiratory system and is closely calibrated to requirements. It can be argued that in adults the “RDA” for O_2_ is determined essentially by the need to maintain energy balance so that intake and expenditure are equal, especially in those of normal body weight. This will depend, of course, on the overall dietary composition in terms of other macronutrients and whether lipids, proteins, or carbohydrates are being oxidised. It will also depend on the level of physical activity as well as the stage in the life cycle, the requirement being higher as with other nutrients during growth, pregnancy and lactation.

## Conclusions

Gaseous oxygen is not framed as a nutrient in nutritional science (1), despite being essential for all aerobic organisms. This primarily reflects the route of entry—the nose and lungs (or gills), rather than the mouth and gastrointestinal tract which is the path by which all other nutrients enter the body in higher animals. In very simple organisms, there is no fundamental difference in the mechanism by which O_2_ and other nutrients are taken up, differentiation occurring only following the evolution of specialised systems for digestion and respiration. At the cellular level in higher animals, there is also no essential distinction between O_2_ and what are conventionally considered as nutrients.

Deficiency of O_2_ on a whole-body basis is apparent in a number of situations, relating either to the specific environmental niche or to particular disease states. Individual tissues may experience a reduced pO_2_–relative hypoxia—either as part of the physiological milieu to which they are customarily exposed, or as a response to specific diseases. In practise, most tissues are relatively hypoxic, and certainly in relation to the O_2_ tension in alveolar blood. There are pervasive adaptations at a cellular level to reduced pO_2_, with a major switch in energy metabolism from mitochondrial respiration and oxidative phosphorylation to anaerobic glycolysis. This involves substantial shifts in the pattern of gene expression, much of which is driven by specific transcription factors, particularly HIF-1.

The concept of the RDA can be applied to O_2_, as with other nutrients, and toxicity is evident in certain circumstances. It is concluded that O_2_ is an important nutrient, and indeed an essential one, that is required in large quantities and which should be considered in conjunction with what are conventionally regarded as nutrients. As such, O_2_ should be firmly viewed as being part of the landscape of nutritional science.

## Coda

In the 4 August 2017 issue of *Science*, a series of Japanese haikus on each element (“Elemental Haiku”) was presented by Mary Soon Lee (82). The haiku for oxygen encapsulates the critical biological importance of this element:

“*Most of me is you*.

*I strive for independence*,

*Fail with every breath*.”

## Author Contributions

The author confirms being the sole contributor of this work and has approved it for publication.

### Conflict of Interest Statement

The author declares that the research was conducted in the absence of any commercial or financial relationships that could be construed as a potential conflict of interest.
